# Paleolimnology and resurrection ecology: The future of reconstructing the past

**DOI:** 10.1111/eva.12556

**Published:** 2017-12-14

**Authors:** David R. L. Burge, Mark B. Edlund, Dagmar Frisch

**Affiliations:** ^1^ St. Croix Watershed Research Station Science Museum of Minnesota Marine on St. Croix MN USA; ^2^ Water Resources Science Graduate Program University of Minnesota St. Paul MN USA; ^3^ School of Biosciences University of Birmingham Birmingham UK

**Keywords:** community ecology, life‐history evolution, natural selection and contemporary evolution, species interactions

## Abstract

Paleolimnologists have utilized lake sediment records to understand historical lake and landscape development, timing and magnitude of environmental change at lake, watershed, regional and global scales, and as historical datasets to target watershed and lake management. Resurrection ecologists have long recognized lake sediments as sources of viable propagules (“seed or egg banks”) with which to explore questions of community ecology, ecological response, and evolutionary ecology. Most researchers consider *Daphnia* as the primary model organism in these efforts, but many other aquatic biota, from viruses to macrophytes, similarly produce viable propagules that are incorporated in the sediment record but have been underutilized in resurrection ecology. The common goals shared by these two disciplines have led to mutualistic and synergistic collaborations—a development that must be encouraged to expand. We give an overview of the achievements of paleolimnology and the reconstruction of environmental history of lakes, review the untapped diversity of aquatic organisms that produce dormant propagules, compare *Daphnia* as a model of resurrection ecology with other organisms amenable to resurrection studies, especially diatoms, and consider new research directions that represent the nexus of these two fields.

## 1 LAKE SEDIMENTS AS ENVIRONMENTAL AND EVOLUTIONARY ARCHIVES

Lakes are a dominant feature on much of the earth's surface with over 117 million lakes greater than 0.2 ha estimated to cover about 3.7% of the land area (Verpoorter, Kutser, Seekell, & Tranvik, 2014). Importantly, almost every lake accumulates sediments at rates of millimeters to 10s of cm per year in conformable patterns of deposition. Paleolimnologists rely on the ability to sample, date, and analyze physical and biogeochemical signals preserved in the sediments to determine lake and landscape evolution, environmental change in lakes, and lake response to local, regional, and global drivers. In contrast to terrestrial sediments that are often disturbed by erosion and bioturbation, aquatic sediments accumulate at measurable rates because they are often buffered from physical, chemical, and biotic disturbance. Because of these features, many different proxies of the sediment record are preserved that can be analyzed with a higher temporal resolution when compared to terrestrial paleontology (Cohen, 2003; Sadler, 1981).

Ingrained in the life history of many organisms adapted to living in lakes are dormancy strategies that result in resistant propagules specialized to survive or perennate at the sediment–water interface. Unsuccessful termination of the dormant phase or entrainment back to the water column can result in propagules being permanently buried in the sediment column. This “egg bank” (Cáceres & Hairston, 1998) has been a rich repository of information for resurrection ecologists.

Resurrection ecology is a fast‐moving field combining evolutionary biology, ecology, and paleobiology to study how terrestrial (Seddon, Moehrenschlager, & Ewen, 2014) and aquatic species (Brendonck & De Meester, 2003) persist, propagate, and evolve under the forces of ecological change. For some, resurrection ecology may suggest de‐extinction by implanting fragments of ancient DNA isolated from fossil tissues into the oocytes of modern organisms; however, this process has not been viably successful (Folch et al., 2009) and is not the focus of this paper. Although many terrestrial organisms produce dormant structures (e.g., seeds), here we focus on freshwater aquatic organisms whose viable dormant propagules coupled with the rich paleo‐limnological sediment record and its high temporal resolution (see above) provide opportunities to study populations, organisms, and environments of the past. Many zooplankton and microbes have evolved unique dormant life stages that survive or persist in lake sediments (Hairston & Fox, 2009). Organisms deposit dormant stages in lake sediments to survive unfavorable growing conditions such as desiccation in ephemeral ponds and overwintering in larger water bodies. After the unfavorable growing conditions pass, bioturbation and wind‐driven turbulence are mechanisms that mix sediments into the water column providing triggers to end dormancy. Cáceres and Hairston (1998) describe the dormant stages at the sediment–water interface that can mix back into the water column as the active egg bank, whereas those permanently trapped in the lake sediments form the inactive egg bank. It is the viable dormant stages of the inactive egg bank that provide a unique source of populations from the past. Resurrection ecologists can bring back to life viable dormant propagules of ancient aquatic organisms that allow phenotypic or genomic characterization rather than piecing together fragmentary fossil DNA. Resurrected organisms provide exceptional opportunities to study evolutionary processes, are a potential source of extinct species or lineages, and can be used to test paleo‐proxies of environmental change (reviewed in Orsini et al., 2013).

The same conditions that allow egg banks to persist in lake sediments—dark, cold, anoxic—are also conducive to preservation of the many physical and biogeochemical signatures or proxies that paleolimnologists use to understand timing and magnitude of ecological change in lakes. Paleolimnology is a well‐established field where a wide variety of abiotic (e.g., bulk density, dry mass, radioactive isotopes, mineralogy) and biotic proxies (e.g., fossils, species abundances, presence/absence, pigments) preserved in the sediments are analyzed to reconstruct ecosystem change at timescales ranging from interannual to decadal to millennial (Cohen, 2003; Last & Smol, 2001). Intact lacustrine sediment cores from 10s of centimeters to 100s of meters in length can be recovered from depositional basins using simple line‐operated devices to drilling rigs (Wright, 1991). To develop date–depth relationships in cores, well‐established dating techniques including radioisotopic (especially ^14^C, ^210^Pb, and ^137^Cs) and stratigraphic (e.g., pollen) methods are used (Appleby, 2001). Careful collection and dating of sediment cores ensure that a conformable sedimentation record has been sampled, and allows the many physical and biogeochemical proxies to be confidently analyzed.

The next step in paleoecological investigations is the analysis of proxies to develop precise reconstructions of ecosystem change; this is the framework of paleolimnology and is fundamental for providing environmental context for resurrection ecology. Physical, geochemical, and biological proxies including organic remains such as diatom, cyanobacteria, and zooplankton subfossils, and their dormant propagules, can be analyzed in lake sediments. The information gleaned by proxy presence/absence, abundance, morphology, and condition hold certain relevance for the paleolimnologist, but the same remains and accompanying data will also provide detailed and crucial paleoenvironmental information needed to interpret depositional environment, ecological setting, and resource dynamics for the resurrection ecologist.

Single proxy analyses are still used in paleolimnology to provide specific stressor identification such as historical deposition of heavy metal mining waste in lakes (Kerfoot, Lauster, & Robbins, 1994). Since the 1980s, paleolimnologists have applied quantitative reconstruction techniques to estimate individual historical lake conditions of interest such as pH, salinity, nutrient levels using diatoms (Bennion, Juggins, & Anderson, 1996; Dixit & Smol, 1994; Fritz, Juggins, Battarbee, & Engstrom, 1991; Ramstack, Fritz, Engstrom, & Heiskary, 2003), and dissolved oxygen using chironomid remains (Brodersen & Quinlan, 2006). Quantitative inference models are typically developed by sampling water quality and surface sediments in many lakes (the training set) that captures a gradient of the environmental variable of interest. Exploratory multivariate techniques are used to identify which environmental factors independently and significantly explain, for example, diatom abundance and distribution in the lakes. Predictive models are developed using techniques such as weighted averaging regression and calibration so a subfossil diatom assemblage can be used to predict, for example, historical salinity (Fritz et al., 1991) and total phosphorus levels (also referred to as diatom‐inferred total phosphorus; Ramstack et al., 2003). Recent criticisms of quantitative inference modeling have resulted in appropriate precautions (Juggins, 2013), such as training set sample size and age appropriateness of the flora (Reavie & Edlund, 2013). Given that multiple factors driving assemblage change, not all of the taxa within a flora are sensitive to the constituent of interest, and different models are not easily transferable (Juggins, Anderson, Ramstack Hobbs, & Heathcote, 2013).

A more powerful approach in paleoecology is multiproxy analysis of cores (Birks & Birks, 2006) to reconstruct past lake and watershed conditions. As its name implies, multiproxy analysis relies on simultaneous analysis of a multiple physical and biogeochemical proxies to develop a more complete understanding of timing and magnitude of ecological change, which might include atmospheric deposition, shifts in habitat structure, lake eutrophication, and resulting food web interactions (Sayer, Davidson, Jones, & Langdon, 2010). For example, in Australian billabongs, multiproxy analysis revealed how the progression of eutrophication impacted diatom, macrophyte, cladoceran and chironomid communities (Davidson, Reid, Sayer, & Chilcott, 2013).

Multiproxy studies can also be extended to include whole basin historical reconstructions. For example, diatoms, fossil algal pigments, phosphorus fractions, and biogenic silica were analyzed in a series of 18 cores from a natural riverine lake bordering Minnesota and Wisconsin (USA). Multiproxy and whole basin techniques (Engstrom & Rose, 2013) were melded to develop a precise historical record of nutrient loading, nutrient availability, lake productivity (biogenic silica), and ecological change in primary producer communities, all linked to documented land‐use changes (e.g., logging, agriculture, urbanization) in the watershed (Edlund, Engstrom, Triplett, Lafrancois, & Leavitt, 2009; Edlund, Triplett, Tomasek, & Bartilson, 2009; Triplett, Engstrom, & Edlund, 2009). In addition to their use as environmental archives of human disturbance, paleolimnologists have used lake sediments to understand the timing of exotic species introductions (Edlund, Taylor, Schelske, & Stoermer, 2000; Hairston, Lampert, et al., 1999), speciation (Theriot, Fritz, Whitlock, & Conley, 2006), and postglacial succession (Engstrom, Fritz, Almendinger, & Juggins, 2000).

Single lake paleolimnological studies have also given way to multilake studies that consider among‐lake variation in ecological response, regional trend assessment, and large‐scale syntheses of stressor impacts. Climate‐mediated shifts in diatom assemblages showed temporal variability and differences in magnitude of change in both regional (Shinneman et al., 2016) and hemispheric scales (Rühland, Paterson, & Smol, 2008). Among‐lake differences in stressor response become very evident in multilake studies. Carbon burial, a proxy for in‐lake productivity and terrestrial C sources, was analyzed in over 100 lakes and showed that ecoregional patterns of land use and development and subsequent nutrient enrichment overwhelmed other potential drivers of carbon sequestration such as climate change (Anderson, Dietz, & Engstrom, 2013; Dietz, Engstrom, & Anderson, 2015). Contrasting patterns of C burial and phosphorus accumulation in two Minnesota lakes were correlated with the lakes’ contrasting development, land use, and lake eutrophic histories. Importantly, these contrasting lake histories provided the ecological framework for interpreting temporal changes in population genetic structure of *Daphnia* based on paleogenetic analysis of dormant eggs (Frisch et al., 2017).

A recent collaboration among paleoecologists posed 50 priority questions to guide the future of paleolimnology (Seddon, Mackay, et al., 2014). Key research directions that were identified included Anthropocene human‐environment interactions, biodiversity and conservation, biodiversity changes over multiple timescales, use of multiple lines of evidence (multiproxy and across spatial and temporal scales), and new developments in paleoecology. Among the questions raised were many that are best answered using resurrection ecology techniques, but more relevant to this paper were the many questions and developments that needed combined efforts from paleolimnologists and resurrection ecologists.

In this review, we explore this key link that is developing between paleolimnology and resurrection ecology. We first discuss the use (to date) of the primary model organism, *Daphnia*, in resurrection ecology, noting both the beneficial and limiting characteristics of this model organism. We then briefly discuss other organisms that produce dormant stages that are deposited into freshwater lacustrine sediments including their relevant life‐history strategies, viability of propagules, and the main utility of each group in paleoecological research. Other organisms that possess dormant stages, such as diatoms, have hardly been utilized in resurrection ecology despite a proven record of utility in paleolimnology as indicators of change in habitat, pH, salinity, and nutrient level. These environmental changes are driven by large‐scale regional and global stressors that are among the strongest selective pressures affecting lakes and their biota (Frisch et al., 2017). We conclude by exploring areas of research that represent the nexus of resurrection ecology and paleolimnology including responses of single species to environmental drivers, assessment of community‐level and multiple organism responses to change, development of better mechanistic understanding of environmental and evolutionary change, and new strategies and technologies available to address these areas.

## 2 *DAPHNIA* SPECIES AS MODEL ORGANISMS IN RESURRECTION ECOLOGY

As the term resurrection ecology was coined (Kerfoot, Robbins, & Weider, 1999; Kerfoot & Weider, 2004), the planktonic crustacean *Daphnia* (water flea) has been developed as a model organism in resurrection ecology. Early studies pioneered experimental research on resurrected *Daphnia* (e.g., Cousyn et al., 2001; Hairston, Lampert, et al., 1999; Hairston, Perry, et al., 1999) to understand evolutionary adaptation to increasing anthropogenic impacts on the environment. In contrast to comparing phenotypic and genomic responses of traits from spatial populations that differ in their environments as well as their genetic background, evolutionary adaptation to environmental change can be directly observed in resurrected temporal populations. Experimental evolution, an alternative to resurrection ecology when resurrected isolates are unavailable, is typically applied to unicellular organisms such as bacteria or yeast or multicellular organisms with short generation times such as *Drosophila* (reviewed in Bell, 2016). However, more recently, experimental evolution using *Daphnia* has gained momentum, with studies of asexually propagated mutation accumulation lines generated over a maximum number of 100 generations (Xu et al., 2012). In contrast, resurrection ecology is geared toward the study of natural *Daphnia* populations shaped by the complexity of the biotic and abiotic environment over timeframes that could span decades or even centuries (Frisch et al., 2014) and thus may represent thousands of asexual generations.

Dormant eggs of *Daphnia* generally result from sexual reproduction except for obligately asexual lineages that occur at higher latitudes (reviewed in Dufresne, Marková, Vergilino, Ventura, & Kotlík, 2011). In *Daphnia*, two eggs are encapsulated together in an ephippium that form egg banks in the sediment of lakes and ponds. Densities of *Daphnia* ephippia deposited in egg banks can reach >10,000 ephippia/m^2^ (Cáceres, 1998; Carvalho & Wolf, 1989). Dormant eggs of *Daphnia* can be hatched for culturing in the laboratory. However, egg viability is impacted by age and environmental conditions of the sediment (Weider, Lampert, Wessels, Colbourne, & Limburg, 1997), limiting the number of hatchlings that can be obtained from several centuries‐old eggs (Morton, Frisch, Jeyasingh, & Weider, 2015). Owing to *Daphnia's* cyclical parthenogenetic life cycle (Decaestecker, de Meester, & Mergeay, 2009), clonal cultures of genetically identical individuals (clonal lineages) can be established in conditions that suppress induction of sexual reproduction and male formation.

To hatch historic eggs from distinct time periods, ephippia are removed from dated sediment, decapsulated and exposed to conditions that induce development. Hatching success is constrained by egg viability and drops with age of the eggs, with >75% hatching from sediment as old as 20 years (Weider et al., 1997) to greatly reduced (≪1%) hatching events in centuries‐old layers (Frisch et al., 2014). Because of this constraint, existing studies are typically focused on resurrected *Daphnia* <70 years old (e.g., Cousyn et al., 2001; Hairston, Lampert, et al., 1999; Hairston, Perry, et al., 1999; Jansen et al., 2017), with some exceptions (120 years (Cáceres, 1998), 600–700 years (Frisch et al., 2014)).

Life‐history experiments of resurrected clonal lineages support the capacity for adaptive responses of *Daphnia* to the environment, for example, to toxic algae (Hairston, Lampert, et al., 1999; Hairston, Perry, et al., 1999), host–parasite interactions (Decaestecker et al., 2009), eutrophication (Frisch et al., 2014), temperature change (Gabriel & Lampert, 1985; Geerts et al., 2015), fish predation (Cousyn et al., 2001), multiple environmental stressors (Orsini, Spanier, & De Meester, 2012), or have provided insight into the evolution of phenotypic plasticity (Henning‐Lucass, Cordellier, Streit, & Schwenk, 2016). The development of high‐throughput DNA sequencing allows phenotypes of resurrected *Daphnia* to be associated with single nucleotide polymorphisms (SNPs) of whole genomes using whole‐genome association studies (GWAS, Miner, De Meester, Pfrender, Lampert, & Hairston, 2012; Orsini et al., 2012), or with transcriptomic patterns of the same resurrected clones (Lack, Weider, & Jeyasingh, 2018; Roy Chowdhury et al., 2015). To identify putative quantitative trait loci (QTLs), resurrected clones with contrasting phenotypes are crossed that represent different time points of the same populations. Such an approach is currently being explored and has recently produced F2 mapping panels for quantitative genetic analyses (L. J. Weider & P. D. Jeyasingh, personal communication).

As detailed above, *Daphnia* species have proven an excellent organismal group to provide the foundation for and expand the field of resurrection ecology. The ability of their dormant eggs to survive centuries, a cyclical parthenogenetic life cycle with short generation times for genetically identical replicates, environmental sensitivity, established genetic and genomic resources, and their easy culturing have made *Daphnia* a model organism leading the field of resurrection ecology. However, use of a single organismal group limits the examination of trophic interactions (Carpenter, Kitchell, & Hodgson, 1985), co‐evolutionary dynamics (Kinnison, Hairston, & Hendry, 2015), and *Daphnia* or other macroinvertebrate communities might not be as sensitive to perturbations as other communities such as diatoms (Justus, Burge, Cobb, Marsico, & Bouldin, 2016).

## 3 OTHER FRESHWATER ORGANISMS WITH DORMANCY STAGES

Ranging from macroinvertebrates, algae, and phages, viable long‐term resting stages have been observed for a wide variety of freshwater organisms. Here, we evaluate the applicability of several freshwater organisms that have also been used as paleolimnological indicators. Based on microbial ecology, Lennon and Jones (2011) characterized propagule dormancy into three stages: initiation, dormancy, and resuscitation. Under this dormancy framework, initiation of asexually produced microbial spores could be triggered by resource limitation or spontaneously; in higher organisms, propagules result from sexual or asexual reproduction in response to biotic cues including predation (Gyllström & Hansson, 2004). Dormancy is associated with physiological changes, energetic costs, and ecological trade‐offs (Lennon & Jones, 2011; Alekseev, De Stasio, & Gilbert, 2007; Shoemaker & Lennon, 2018), and its duration can range from weeks to decades or even centuries (Alekseev et al., 2007). Resuscitation of dormant cells, like initiation, is triggered by environmental cues (Gyllström & Hansson, 2004; Sicko‐Goad, Stoermer, & Kociolek, 1989) or in the case of microbes, dormancy release can also be spontaneous (Lennon & Jones, 2011). We review the dormancy strategies and paleoecological significance of freshwater organisms that are commonly used in paleolimnology and that include dormant propagules as part of their ecology and life history (Table 1). For greater detail on resting stage cytology, metabolism, and dispersal see Ellegaard and Ribeiro (2017) for phytoplankton, and Gyllström and Hansson (2004) and Alekseev et al. (2007) for aquatic invertebrates.

**Table 1:** Distribution of dormant propagules among freshwater organisms including mode of production, viability, trigger for dormancy, conditions for resuscitation of propagules, and paleo‐indicator value

Taxonomic group	Dormant Propagule	Life history	Viability (years)	Dormancy Trigger	Resuscitation	Paleo‐Indicator Value
Cladocera	Ephippium (1)	Sexual, parthenogenesis (1)	600 (2)	Resource limitation, seasonality, crowding, predation (1)	Temperature, photoperiod, dissolved oxygen (1)	Climate (5), lake level (6), trophic state (7), acidification (8), species invasion (9)
Copepoda	Diapausing eggs (1)	Sexual (1)	300 (3)			Lake level (10)
Rotifera	Diapausing eggs (1)	Sexual, parthenogenesis (1)	40 (4)			Eutrophication (11)
Ostracoda	Diapausing eggs (12)	Sexual (12)	n/a	Temperature, photoperiod (12)	Favorable growth conditions (13)	Salinity, precipitation, temperature (14)
Porifera	Gemmule (16)	Parthenogenesis (16)	25 (17)	Osmotic pressure (18)	Temperature (18)	Alkalinity (18), salinity (19)
Cyanobacteria	Akinete (20)	Vegetative (20)	64 (21)	Temperature (22)	Temperature, photoperiod (23)	Eutrophication, temperature (24)
Dinoflagellates	Resting spore (25)	Sexual (25)	90 (26)	End of growing conditions (27)	Photoperiod (28)	Land‐use changes (29)
Diatoms	Resting cell (30)	Vegetative (30)	100 (31)	Temperature, nutrients (30, 32)	Temperature, photoperiod (30, 34)	Acidification (37), salinity (38), eutrophication (39), climate change (40), species invasion (41)
Diatoms	Resting spore (30)	Vegetative (30)	1 (35)	Nutrients (36)	Nutrients (36)	

References in parentheses: (1) Gyllström and Hansson (2004); (2) Frisch et al. (2014); (3) Hairston et al. (1995); (4) Marcus et al. (1994); (5) Smol et al. (2005); (6) Hyvärinen and Alhonen (1994); (7) Bos and Cumming (2003); (8) Nilssen and Sandoy (1990); (9) Keilty (1988); (10) Borromei et al. (2010); (11) Findlay et al. (1998); (12) Hairston et al. (1990); (13) McLay (1978); (14) Xia et al. (1997); (15) Rasmont (1954); (16) Harrison (1974); (17) Simpson and Fell (1974); (18) Harrison and Harrison (1979); (19) Cumming et al. (1993); (20) Miller and Lang (1968); (21) Livingstone and Jaworski (1980); (22) Li et al. (1997); (23) Rengefors et al. (2004); (24) Kling (1998); (25) Dale (1983); (26) Lundholm et al. (2011); (27) Heiskanen (1993); (28) Anderson et al. (1987); (29) McCarthy and Krueger (2013); (30) McQuoid and Hobson (1995); (31) Härnström et al. (2011); (32) Lund (1954); (33) Nipkow (1950); (34) Sicko‐Goad et al. (1986); (35) Garrison (1979); (36) Jewson et al. (2008); (37) Camburn and Charles (2000); (38) Fritz et al. (1991); (39) Stoermer et al. (1996); (40) Boeff et al. (2016); (41) Edlund et al. (2000).

**Figure 1:**
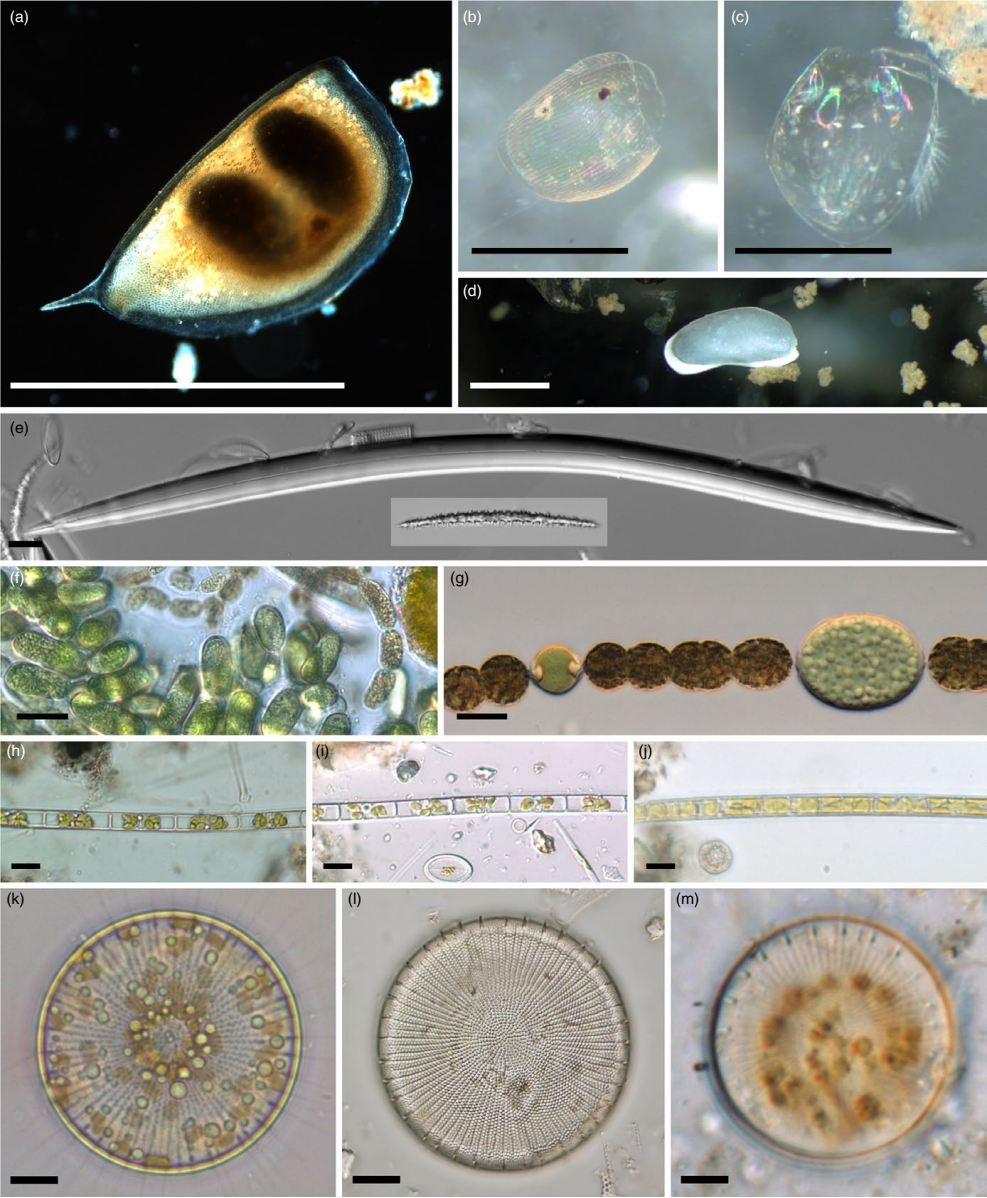
Light micrographs of dormant propagules and sediment microfossils. (a) *Daphnia pulicaria* ephippium with two dormant eggs, (b, c) cladoceran exoskeletons, (d) ostracod shell, (e) freshwater sponge megasclere with smaller microsclere spicule (inset), (f) the cyanobacterium *Dolichospermum* sp. with mass production of akinetes, (g) short filament of *Dolichospermum* sp. showing translucent heterocyte and larger akinete, (h, i) filaments with dormant cells of the diatom *Aulacoseira ambigua,* (j) rejuvenated cell of *A. ambigua,* (k) live vegetative cell of the diatom *Stephanodiscus niagarae,* (l) single valve of *S*. *niagarae* preserved in sediment, (m) dormant resting cell of *S*. *niagarae*. Scale bars = 10 μm (e–m); 1 mm (a, d); 0.5 mm (b, c)

Many species of freshwater zooplankton undergo dormancy as a life‐history strategy and produce resistant propagules for community resilience and dispersal which enables them to survive digestion or transport by birds (Frisch, Green, & Figuerola, 2007; Fryer, 1996). Gyllström and Hansson (2004) provide an extensive review on Cladocera, Copepoda, and Rotifera dormancy, organisms that are also commonly used in paleolimnology (Smol, Birks, & Last, 2001). A discussion on *Artemia* as a suitable organism for resurrection ecology is discussed in this issue (Lenormand et al., 2018). The dormant eggs of calanoid copepods have provided a resource for resurrection ecology that several studies have taken advantage of (e.g., Derry, Arnott, & Boag, 2010; Hairston, Van Brunt, Kearns, & Engstrom, 1995; Jiang et al., 2012). Zooplankton generally produce dormant propagules sexually, as fertilized diapausing eggs; however, Cladocera and Rotifera can also produce dormant propagules parthenogenetically. Dormant eggs of the Anomopoda, a suborder of Cladocera which includes *Daphnia,* are enveloped in a protective chitinous structure known as an ephippium (Figure 1a). Gyllström and Hansson (2004) summarized a variety of abiotic and biotic trigger mechanisms for inducing dormant egg production in zooplankton including resource limitation, seasonality, crowding, and predation. Zooplankton propagules have an increasingly well‐documented record of longevity in lake sediments. Frisch et al. (2014) hatched *Daphnia* eggs that were dormant in lake sediments for up to ~600–700 years. Hairston et al. (1995) found dormant calanoid copepod eggs to be viable for over 300 years. Rotifera appear to have a shorter dormant egg longevity of 35–40 years (Marcus, Lutz, Burnett, & Cable, 1994; Nipkow, 1961). In all studies reviewed (*n* = 49), Gyllström and Hansson (2004) found that environmental cues (temperature, light, dissolved oxygen) most often associated with seasonality could trigger resuscitation of cladoceran, copepod, and rotifer propagules. Hatching rates of dormant zooplankton propagules are negatively correlated with sediment age and significantly inhibited by environmental stressors such as metal contamination (Rogalski, 2015).

Apart from dormant propagules, most zooplankton also produce physical remains that are incorporated into the sediment record and are used by paleoecologists. Cladocera have a chitinous exoskeleton (Figure 1b,c), which allows body parts such as carapaces, claws, and spines to persist in lake sediments. Cladoceran remains from lake sediments have been used to track changes in climate (Lotter, Birks, Hofmann, & Marchetto, 1997; Smol et al., 2005), lake level (Hyvärinen & Alhonen, 1994), trophic state (Bos & Cumming, 2003; Boucherle & Züllig, 1983), lake acidity (Nilssen & Sandoy, 1990), and non‐native species invasion (Keilty, 1988). Copepod exoskeletons remain in the lake sediment records, but do not preserve as well compared with Cladocera (Rautio, Sorvari, & Korhola, 2000), therefore copepod diapausing eggs (Bennike, 1998) and spermatophores have been suggested as useful paleolimnological indicators of lake level (Borromei et al., 2010). Copepod spermatophores have also been used in conjunction with zooplankton community data for reconstructing anthropogenic eutrophication (Findlay, Kling, Rönicke, & Findlay, 1998). Rotifers are primarily identified from lake sediments by their loricas. Rotifer loricas have been used to reconstruct early postglacial community succession (Swadling, Dartnall, Gibson, Saulnier‐Talbot, & Vincent, 2001) and nutrient dynamics of lakes (Findlay et al., 1998; Turton & McAndrews, 2006).

In addition to Cladocera, other Branchiopoda that form diapausing eggs include Anostraca, Conchostraca, Mysida, Notostraca, and Spinicaudata (Fryer, 1996). Anostracan remains have been used to reconstruct lake salinity (Bos, Cumming, & Smol, 1999); however, these groups are not commonly used in paleoecological reconstructions or resurrection ecology.

Ostracods are a group of freshwater microcrustaceans commonly used in paleolimnology and that form diapausing eggs. Hairston, Dillon, and De Stasio (1990) found that diapause initiation in ostracods occurred during the winter in response to temperature and photoperiod reduction. Ostracod diapause eggs are resistant to freezing (Theisen, 1966) and desiccation (McLay, 1978). McLay (1978) found that when favorable growth conditions returned, some species would develop immediately whereas other species underwent a prolonged dormancy period. In paleolimnology, ostracod abundance determined from light microscopic identification of remnant shells (Figure 1d) has been used to reconstruct fluctuations in lake level (Viehberg, 2004) and gradients of ionic concentrations in modern limnology (Van der Meeren, Almendinger, Ito, & Martens, 2010). Ostracod shell chemistry is an especially powerful tool in paleolimnology. Isotope composition of ostracod shells directly reflects water chemistry at the time of shell formation so shell chemistry has been used to reconstruct paleosalinity, precipitation, and temperature (Xia, Haskell, Engstrom, & Ito, 1997). While ostracod eggs have not been reported from deep‐water sediment cores, they have been collected and hatched from ponds (Rossi, Albini, Benassi, & Menozzi, 2012).

Members of the Porifera, the freshwater sponges, produce dormant stages known as gemmules (Rasmont, 1954). Gemmule formation is parthenogenetic and can be triggered by increasing osmotic pressure; increasing temperature can induce gemmule hatching (Simpson & Fell, 1974). Light, season, and nutrient availability influence the size and thickness of gemmules, which determines their resilience. Porifera gemmules have been found to be viable from 25‐year‐old lake sediments (Harrison, 1974). Freshwater sponges deposit two types of siliceous spicules; microscleres are smaller and used for species identification, whereas megascleres are enumerated for population estimates (Harrison, 1988). Freshwater sponges have been used as indicators of alkalinity (Harrison & Harrison, 1979) and paleosalinity (Cumming, Wilson, & Smol, 1993), and the density and thickness of spicules have been related to silica dynamics (Jewel, 1939; Kratz, Frost, Elias, & Cook, 1991).

A wide variety of unicellular organisms are known to form dormant stages in lake sediments including phages, bacteria, cyanobacteria, dinoflagellates, and diatoms. Lennon and Jones (2011) provide a comprehensive review of the diversity of initiation, dormancy, and resuscitation found in heterotrophic bacteria; however, they did not examine cyanobacteria in detail. Cyanobacteria are photosynthetic bacteria and many taxa vegetatively form dormant propagules called akinetes (Figure 1f,g), which are fortified cells that can survive desiccation and adverse growing conditions (Livingstone & Jaworski, 1980; Miller & Lang, 1968; Yamamoto, 1975). Li, Watanabe, and Watanabe (1997) found that decreasing temperature was the primary factor inducing akinete formation. In laboratory experiments, cyanobacteria failed to produce akinetes in the dark, and akinetes become significantly less viable when exposed to temperatures below 20°C (Agrawal & Singh, 2000). Akinetes have been resuscitated from 64‐year‐old lake sediments (Livingstone & Jaworski, 1980). Along with resuspension of lake sediments, environmental cues for the resuscitation of akinetes include increasing temperature and light (Rengefors, Gustafsson, & Ståhl‐Delbanco, 2004). Cyanobacteria produce microfossils and pigment signatures in sediments that are used for paleoecological inference (Kling, 1998; Leavitt & Hodgson, 2002). Akinetes from lake sediments are used as a proxy for cyanobacteria abundance; Kling (1998) interpreted increasing cyanophytes as a paleo‐proxy for increasing temperature and phosphorus. Taranu et al. (2015) used pigment concentrations obtained from 108 sediment cores to demonstrate increasing abundances of cyanobacteria as a response to eutrophication and climate change.

Preserved alongside cyanobacterial remains in sediments are viable cyanophages, viruses that infect and lyse cyanobacteria (Hargreaves, Anderson, & Clokie, 2013). Although virus‐like particles are not used in paleoecological analyses, their utility in resurrection ecology to study predator‐prey evolutionary relationships has been explored. Viable cyanophages were isolated from sediments up to 50 years old and used to infect cultures of the cyanobacterium *Microcystis* (Hargreaves et al., 2013).

Dinoflagellates are unicellular motile algae most commonly associated with red tides in coastal marine waters, but they are also common freshwater phytoplankton. Some species are multitrophic and able to switch between autotrophy, herbivory, and parasitism. Many species are surrounded by an armored theca of cellulose plates (Carty, 2003). The dormant propagule produced by dinoflagellates is a cyst resulting from sexual reproduction (Dale, 1983). Dinoflagellates appear to undergo encystment after blooming as part of the annual life cycle (Heiskanen, 1993). The dinoflagellate cyst must undergo a period of maturation before hatching, which can be up to 5 months long (Binder & Anderson, 1986). Lundholm et al. (2011) resuscitated dinoflagellate cysts in lake sediments that were dated 90 years old. Germination of dinoflagellates appears to be induced by light exposure and suppressed by anaerobic conditions (Anderson, Taylor, & Armbrust, 1987). In paleolimnology studies, dinoflagellate resting cysts are enumerated in sediments using light microscopy (Livingstone, 1984). Using cyst abundances, the community composition of dinoflagellates reflected land‐use changes by indigenous people and European settlers in Ontario (McCarthy & Krueger, 2013; McCarthy et al., 2011).

Diatoms are photosynthetic microalgae and are the most diverse group of algae (Round, Crawford, & Mann, 1990). Among the microalgae, they are characterized by their ornamented two‐part cell wall composed of biogenic silica (Figure 1k,l). Diatoms produce two types of dormant vegetative stages including “resting spores” and “resting cells” (Kaczmarska et al., 2013; McQuoid & Hobson,^1996^). Dormant spores are morphologically distinct and more heavily silicified than vegetative cells and are typically formed in response to decreasing nitrate (Davis, Hollibaugh, Seibert, Thomas, & Harrison, 1980; Kuwata & Takahashi, 1990) and phosphate concentrations (Jewson et al., 2008). Diatom spore production is particularly common in coastal marine diatoms, although a few freshwater genera also form spores (Edlund & Stoermer, 1993; Edlund, Stoermer, & Taylor, 1996). Dormant cells (Figure 1h,i,m) are formed in response to several environmental triggers including lower temperature and light conditions (Lund, 1954) and silica limitation following lake stratification. Silica limitation and stratification trigger diatoms to increase their sinking rate and shift their physiology to increased storage of carbohydrates and lipids and condensation of cell organelles around the nucleus (Gibson & Fitzsimons, 1990; Gibson & Foy, 1988; Sicko‐Goad et al., 1989). It has also been suggested that some cold‐favoring diatoms initiate dormant cells in response to warming temperatures (Nipkow, 1950). While dormant spores have shorter viability, on the order of weeks (Hargraves & French, 1975) to a year (Garrison, 1979), diatom dormant cells are viable for many decades while persisting in lake sediments (Sicko‐Goad, Stoermer, & Fahnenstiel, 1986; Stockner & Lund, 1970). Dormant spores excyst under experimental nutrient replenishment (Jewson et al., 2008). Diatom dormant cell resuscitation appears to be less affected by nutrient levels (Hollibaugh, Seibert, & Thomas, 1981), rather greater recruitment occurs under increased light and temperature regimes (Figure 1j; Lund, 1954; Sicko‐Goad et al., 1986; McQuoid & Hobson, 1995). The deposition and preservation of diatoms have enabled paleolimnologists to reconstruct historical changes in lake acidity (Battarbee, 1991; Camburn & Charles, 2000), salinity (Cumming, Wilson, Hall, & Smol, 1995; Fritz et al., 1991), cultural eutrophication (Battarbee, 1978; Reavie, Hall, & Smol, 1995; Schelske, Conley, Stoermer, Newberry, & Campbell, 1986; Stoermer, Emmert, Julius, & Schelske, 1996), climate change (Boeff, Strock, & Saros, 2016; Kilham, Theriot, & Fritz, 1996; Saros et al., 2012), and species invasion (Edlund et al., 2000).

It is obvious that planktonic organisms dominate this list; however, an equal or even greater diversity of organisms inhabits the littoral zone of lakes and may similarly utilize dormant propagules. Paleolimnologists recognize that littoral zone diversity is not fully represented in deep‐water sediment cores and vice versa. They also recognize that littoral habitats are less suited for paleolimnology because of noncomforable sedimentation, loss of temporal resolution, and higher rates of grazing, resuspension, and mineralization that make littoral habitats less suitable for preserving resting stages (Anderson, 2014).

With respect to freshwater organisms that include a period of dormancy in their life cycle, many studies have focused on ecology, physiology, and length of dormancy. From a paleoecological perspective, most of these taxa have also been readily adopted as valuable indicators of ecological change. Research linking dormancy ecology and longevity with paleoecology has been limited to only a few model organisms, primarily the cladoceran *Daphnia*. Few other organisms have been systematically investigated for their potential as models in resurrection ecology, but several offer strong potential because of their prevalence across environmental and temporal scales. One limiting factor to resurrection ecology that can occur in copepods, ostracods, porifera, and dinoflagellates is that the dormant propagules are produced sexually. If there were functional phenotypes selected by the environment, that information could be lost during the genetic recombination occurring during resting stage formation. This would make the relationships between hatched organisms and historical environmental cues more difficult to detect. A further hindrance can be that viable dormant propagules cannot be successfully resuscitated from lake sediments, such as ostracods. Cyanobacteria and diatoms both produce vegetative resting stages, and these stages will be phenotypically identical to historic populations. With greater understanding of the phenotypic–environmental relationships, hatching biases, and further paleoecological associations, these organisms will likely serve as suitable model organisms for resurrection ecology studies.

## 4 DIATOMS AS THE NEXT MODEL ORGANISM IN RESURRECTION ECOLOGY

With a growing body of literature on paleolimnological indicator values, life histories, phylogenies, dormant stages, and advances in molecular analysis, diatoms are a strong candidate to become a model for resurrection ecology. Diatoms are a diverse group of unicellular or colonial photosynthetic organisms that are unique by possessing an opaline silica cell wall called a frustule (Round et al., 1990). The frustules are intricately ornamented, and when viewed in the light microscope, species‐level identifications can be readily made. The silica cell walls may persist for millions of years in sediments with a sufficiently high abundance to reconstruct entire life histories (Jewson & Harwood, 2017). With global estimates of over 100,000 species (Mann & Droop, 1996), which occupy most marine and freshwater habitats, diatoms have proven useful as biological indicators of environmental change among many aquatic systems (Smol & Stoermer, 2010).

Whereas detailed life history and ecological observations for many taxonomic groups of diatoms are wanting, *Aulacoseira* Thwaites is a freshwater planktonic diatom genus that has been well studied. *Aulacoseira* is a species‐rich genus and among the most ancestral class of diatoms (Coscinodiscophyceae; Theriot, Cannone, Gutell, & Alverson, 2009). Edgar and Theriot (2004) established phylogenetic relationships of 45 species based on morphology. Diversity in the genus is still being discovered; at least 15 new species have been recently described or transferred (English & Potapova, 2009; Houk, 2007; Morales, Rivera, Dc Rubin, Vis, & Houk, 2015; Novelo, Tavera, & Ibarra, 2007; Pearce, Cremer, & Wagner‐Cremer, 2010; Tanaka, Nagumo, & Akiba, 2008; Tremarin, Ludwig, & Torgan, 2012, 2014; Tremarin, Paiva, Ludwig, & Torgan, 2013; Usoltseva & Tsoy, 2010; Van de Vijver, 2012). Given such high species richness, *Aulacoseira* species are found from low‐gradient eutrophic (Leland & Porter, 2000) to oligotrophic high‐gradient montane rivers (Morales et al., 2015), deep oligotrophic (Jewson & Granin, 2015) to shallow eutrophic lakes (Davey, 1987; Gibson, Anderson, & Haworth, 2003), and even in riverine estuaries (Wang et al., 2009). *Aulacoseira* spans a latitudinal/temperature gradient from lakes in the tropics (Tremarin et al., 2013) to the northern boreal forests (Fallu, Allaire, & Pienitz, 2000). *Aulacoseira* also appear to be highly adaptable to environmental change; many paleolimnological studies document shifts in relative abundance of *Aulacoseira* species, but not extirpation, in response to eutrophication and acidification (e.g., Edlund, Engstrom, et al., 2009).

In addition to biogeographical and ecological characterization, the life histories of several *Aulacoseira* species have been well studied. Jewson (1992) observed the phenology of a population of *Aulacoseira subarctica* (O. Müller) Haworth. He found that *A. subarctica* settled to the lake bottom as dormant cells during the summer and resumed population growth after being resuspended into the plankton during autumn turnover. Once light intensities decreased, sexual reproduction was induced in a small portion of the population during the winter to create annual cohorts. Edlund and Stoermer (1997) characterized this reproductive strategy in diatoms as asynchronous sexuality under good growth conditions. Jewson, Khondker, Rahman, and Lowry (1993) observed similar auxosporulation conditions in *A*. *herzogii*. Jewson (1992) concluded that a full life cycle of an *A. subarctica* cohort could take from 15 to 100 years; however, environmental and physiological controls resulted in a life cycle completed every 4–6 years. He also noted that during times of unfavorable conditions dormant stages were common in his study population of *A. subarctica*.

Dormant cells were first documented by Nipkow (1950) in *Melosira islandica* ssp. *helvetica* (now *Aulacoseira helvetica*) and by Lund (1954, 1955) in *M. italica* ssp. *subarctica* (now *A. subarctica*). Resting cells were subsequently reported for *A. granulata* (Sicko‐Goad et al., 1986), *A. skvortzowii* (Jewson et al., 2008), and *A. baicalensis* (Jewson & Granin, 2015; Jewson, Granin, Zhdarnov, Gorbunova, & Gnatovsky, 2010). Dormant stages appear to have long been present in *Aulacoseira* life history as noted in species from the middle Eocene (Wolfe, Edlund, Sweet, & Creighton, 2006).

Stockner and Lund (1970) were the first to resurrect *Aulacoseira* dormant cells from sediments 11 and 15 cm deep in cores taken from several English Lake District lakes. They estimated that dormant cells in the sediments might be viable for up to 50 years. Sicko‐Goad et al. (1986) resuscitated diatoms from sediments and documented changes in cell condition, cellular ultrastructure, and reactivation of chloroplast function during the rejuvenation process. Their experiments confirmed in situ observations that aphotic conditions combined with decreasing temperature induced dormant cell formation in *Aulacoseira*. McQuoid, Godhe, and Nordberg (2002) resurrected diatoms from over 37‐year‐old sediments in a Swedish fjord, laying the foundation for a study of the oldest known diatom resurrection. Härnström, Ellegaard, Andersen, and Godhe (2011) conducted diatom resurrection ecology from sediments in a Danish fjord. They resurrected 100‐year‐old *Skeletonema marinoi* resting cells from maritime fjord sediments in Denmark. Dormant cells were resuscitated and cultured for molecular analyses that included sequencing one rRNA and two internal transcriber genes. The greatest genetic difference observed was between open water and fjord samples rather than related to trophic changes within the fjord.

Diatoms have been cultured to answer a wide variety of ecological questions including investigations on cytology (Schmid, 2001; Sicko‐Good, Simmons, Lazinsky, & Hall, 1988), pesticide inhibition (Guanzan & Nakahara, 2002), reproduction (Basu et al., 2017), environmental mesocosms (Saros, Michel, Interlandi, & Wolfe, 2005), and ontogeny (Schmid, 1979). Several laboratory culture experiments have been conducted with *Aulacoseira* with resting cell ecology and development. As phosphorus and silica become limiting and cellular growth slows, diatoms respond by increasing their sinking rates (Gibson, 1984) to expedite their journey to the lake sediments and avoid grazing by zooplankton. In culture, Gibson and Foy (1988) found that when silica and phosphorus became limiting, *A. subarctica* allocates resources to increasing lipid storage. Gibson and Fitzsimons (1990) found that under aphotic conditions, *A. subarctica* initially utilized carbohydrates followed by lipids and after a month of darkness, cells would initiate resting stages. Gibson and Fitzsimons (1991) found that light interruptions of aphotic periods had adverse effects on cell growth. For a riverine population of *A. granulata*, laboratory experiments suggested that resting cell resuscitation was initiated by the presence of another alga *Gloeocystis planctonica* (Poister, Schaefer, Baert, Tracey, & Richards, 2015).

Recent work with diatom cultures has included the identification of functional parts of the genome. Only a few diatom species have fully sequenced genomes and the genomes appear to be relatively small at 34 Mbp (e.g., *Thalassiosira pseudonana* Hasle & Heimdal; Armbrust et al., 2004), 44–62 Kbp (e.g., *T. pseudonana*; Armbrust et al., 2004; *Seminavis robusta* Danielidis & Mann; Brembu et al., 2014; *Skeletonema marinoi* Sarno & Zingone; An, Kim, Noh, & Yang, 2016; *Asterionella formosa* Hassall; Villain et al., 2017), and 129–151 Kbp (e.g., *T. pseudonana;* Armbrust et al., 2004; *S. robusta*; Brembu et al., 2014) for the nuclear, mitochondrial, and chloroplast genomes, respectively. The availability of full genomes has allowed researchers to determine gene function using transcriptomics and proteomics studies (Di Dato et al., 2015; Muhseen, Xiong, Chen, & Ge, 2015). For example, in *T. pseudonana*, transcription of genes relating to silica acquisition was up‐regulated in preparation for rapid recovery during silica limitation (Shrestha et al., 2012). Furthermore, examination of transcripts suggested that *T. pseudonana* has multiple methods of phosphorus acquisition and allocation to cope with variable concentrations and forms of phosphorus (Dyhrman et al., 2012). Nunn et al. (2013) found cells could switch metabolic pathways to internally recycle nutrients, therefore reducing the effect of environmental resource limitation.

While microorganisms can be difficult to work with, two methods have been developed recently that facilitate culturing and genomics studies on diatoms. The use of serial dilution enables greater ease of extracting and resuscitating dormant resting cells from lake sediments (Piredda et al., 2017). In addition to more efficient culturing of resting cells, single cell isolation and DNA extraction have proven successful for nuclear and chloroplast genomics (Lefebvre, Hamilton, & Pick, 2017; Pinseel et al., 2017). PCR amplification of DNA from single cells or dormant eggs could provide a suitable amount of genetic material from a small amount of source material for paleogenomic studies to forgo resurrection and culturing (Frisch et al., 2017; Hamilton, Lefebvre, & Bull, 2015).

Diatoms are prime candidates to become model organisms for resurrection ecology. Diatoms are globally significant primary producers, form the base of many aquatic foodwebs, and are characterized by high population numbers, diversity, turnover, and longevity in lake sediments, which allows diatoms to respond rapidly to environmental changes and leave highly informative sediment records. Further, the number of taxa that form dormant cells is large; Sicko‐Goad et al. (1989) identified 17 diatom species forming dormant resting cells in the Laurentian Great Lakes. There is a growing literature on the use of diatoms for in situ ecological and life‐history studies. This is because they are highly adaptable to experimental culturing, and their strong relationship to paleo‐limnological environments (Jewson, 1992; Saros et al., 2005). *Aulacoseira* has a strong foundation in paleolimnological, resting cell, and molecular studies; future transcriptomic and proteomic studies on cell function would increase their utility in future resurrection ecology studies. Finally, diatoms have a rich history in modern‐ and paleo‐limnological literature combined with the life‐history trait of dormant cell formation and a growing genomic understanding that can expand their use in resurrection ecology to studying topics such as the effects of eutrophication or climate change on primary producer and primary consumer relationships or rapid evolution within the phytoplankton.

## 5 A MARRIAGE OF NECESSITY

Reiterating the statement by Pelletier, Garant, and Hendry (2009) that “nothing in evolution or ecology makes sense except in the light of the other,” we propose that a close collaboration between the fields of paleolimnology and resurrection ecology is important and inevitable. Carefully reconstructed environmental histories of entire lake ecosystems provide a powerful framework to interpret the ecological and evolutionary responses of resurrected organisms in the context of the environment in which they evolved. Similarly, to move paleolimnology beyond a descriptive science and to test ecological and evolutionary hypotheses based on its findings, experimental work is required that can be delivered by resurrection ecology. While studies in resurrection ecology with *Daphnia* have already been fruitful, there is a suite of other organisms ready to be awakened to test predictions across an array of taxonomic diversity. The combination of paleolimnology and resurrection ecology will be key to empirically provide answers to questions surrounding the capacity of organisms to adapt to rapid environmental change, one of the most pressing problems that our planet faces today.

### 5.1 Evolutionary responses to environmental change

The loss of species from lakes is often documented in paleolimnology, but is less commonly approached via resurrection ecology. There are hundreds of paleolimnological studies that apply correlative analyses to document and hypothesize why aquatic species become locally extirpated. For example, Stoermer, Wolin, Schelske, and Conley (1985) documented the local extirpation of the deep chlorophyll layer diatom community (numerous *Cyclotella* diatom species) following post‐European eutrophication of Lake Ontario. The combined effects of light and silica limitation following enhanced nutrient loading resulted in the loss of this characteristic diatom community from the lake.

Examples of species extirpation studied with resurrection ecology are less numerous. By analyzing ephippia in sediment cores, Hairston, Lampert, et al. (1999) and Hairston, Perry, et al. (1999) discovered that *Daphnia exilis* had been introduced into Lake Onondaga in the 1920s and persisted into the 1980s. Based on genetic analysis of resurrected populations from the 1970s and 1980s, it was determined that a single genotype had been introduced into the lake, and that at the time of its local extirpation the population had remarkably low genetic diversity that may have contributed to population demise.

Whether a species is lost from a habitat is determined by their adaptive capacity; does their phenotypic plasticity allow them to respond to changing abiotic and biotic pressures, or if not, can they disperse, undergo diapause or more rapidly evolve simultaneous with environmental change (Reed, Schindler, & Waples, 2011)? Adaptive capacity is one way to examine an organism's ability to change niche space (Beever et al., 2015; Chevin, Lande, & Mace, 2010). The adaptive capacity model estimates a species’ chance of survival or extinction by evaluating the costs of phenotypic plasticity and overall genetic variance against the magnitude and duration of an environmental stressor. The rate of environmental change and species adaptation is key in determining the potential for survivability (Hairston, Ellner, Geber, Yoshida, & Fox, 2005). To understand the adaptability of species and to improve niche models, species resurrection must be coupled with a detailed knowledge of specific environmental change on long timescales in natural settings, a deliverable product of paleolimnological research. This will ultimately bring a more complete understanding of how current environmental pressures might affect biodiversity and evolution (Franks, Hamann, & Weis, 2018).

### 5.2 Eco‐evolutionary dynamics of biological communities

While understanding how a single species might adapt to environmental perturbation, it is also important to understand how the interplay of community responses can affect evolutionary dynamics. Community‐level responses have often been observed in paleolimnology. The abundance of diatom and chironomid assemblages has been used to reconstruct Holocene climate changes (Reinemann, Porinchu, Bloom, Mark, & Box, 2009). Also, diatoms, chrysophytes, and cladocerans have been used to reconstruct lake acidity (Arseneau, Driscoll, Brager, Ross, & Cumming, 2011). However, the interplay among members of these paleo‐communities and their community response to the environment has not been directly assessed, and the drivers of population change may be more complicated than expected (Becks, Ellner, Jones, & Hairston, 2012; Kinnison et al., 2015).

In some cases, eco‐evolution can be more strongly driven by interspecies evolution rather than environmental change or resource availability, as shown in predator‐prey relationships (Becks et al., 2012). Evolutionary feedback loops can exist that are not readily observed in assemblage data (Kinnison et al., 2015). Dormant propagules in lake sediments offer the unique ability to use natural, long‐term community records, whose propagules can be resuscitated for experimentation, to understand models of community adaptation and eco‐evolutionary dynamics.

### 5.3 A mechanistic approach links paleoecology and resurrection ecology

In paleolimnology one often unspoken and untested assumption in the interpretation of sediment records is that the ecology of organisms was the same in the past as it is today. We interpret the ecology of the past using our knowledge of modern ecology, which rarely accounts for species adaptation. For example, Stoermer, Emmert, and Schelske (1989) documented size decrease in the diatom *Stephanodiscus niagarae* during cultural eutrophication in Lake Ontario and suggested that the diatom might be succumbing to sexual failure. However, Edlund and Stoermer (1991) showed that small‐celled populations of *S*. *niagarae* from eutrophic areas of the Great Lakes were perfectly capable of undergoing sexual reproduction, a phenotypic life‐history adaptation to nutrient enrichment. While most paleolimnologists examine only sediment records of species response and change, recent studies link historical species responses to modern experimental species responses (Saros, 2009). Saros et al. (2005) conducted experiments relating the response of modern diatom species to nutrient supply and then interpreted the historical response of the same taxa in alpine lakes using the experimental results. Mechanistic studies have also been applied to resurrection ecology with the help of molecular tools. Common garden experiments (Kerfoot & Weider, 2004) have been used on resurrected *Daphnia* to genetically characterize historical populations as they adapted to predation. Roy Chowdhury et al. (2015) used resurrected *Daphnia* from a eutrophic Minnesota lake to study adaptation using a transcriptomics approach to compare gene expression under different food quality scenarios. As we expand the database of diatom genome sequences, changes in genes and proteins can be related to cell functionality to better understand historical species response (Davis, Shaw, & Etterson, 2005).

### 5.4 Paleoecology, resurrection ecology and restoration ecology

The resuscitation of dormant propagules from the sediment “seed bank” may allow organisms specific to each lake to recolonize under restored conditions and could prove useful for re‐establishing the lower trophic levels during restoration efforts and help preserve the Earth's remaining biodiversity. Ecological monitoring and environmental policies in the late 20th century have led to the rehabilitation of some lakes from effects of acidification (Lotter, 2001; Stoddard et al., 1999) and cultural eutrophication (Antoniades et al., 2011). However, after a successful return to historical water quality conditions, a novel ecosystem usually results, that is, communities do not return to their historical makeup (Lotter, 2001). In part, restoration efforts may be confounded by alternative or multiple environmental drivers such as climate change (Battarbee et al., 2011; Sivarajah, Rühland, & Smol, 2017) resulting in extended periods of recovery or a moving baseline for restoration targets (Battarbee, Anderson, Jeppesen, & Leavitt, 2005; Bennion, Battarbee, Sayer, Simpson, & Davidson, 2011). Alternatively, modern populations may not have the adaptive capacity to reorganize to similar historical communities. One common practice in ecological restoration is the facilitated recolonization by similar communities, ideally with similar phenotypes (Buisson, Alvarado, Le Stradic, & Morellato, 2017). Whereas plant (Ozimek, Gulati, & van Donk, 1990) and fish communities (Søndergaard, Lauridsen, Johansson, & Jeppesen, 2017; Volta, Yan, & Gunn, 2016) are commonly the focus of restoration efforts, communities that produce dormant propagules such as zooplankton, phytoplankton, and bacterial communities, are often neglected. While bio‐manipulation has often yielded unintended consequences, suggested strategies for facilitating resurrection in terrestrial ecosystems can be applied to successful restoration of extirpated aquatic taxa. The resting stages of phytoplankton can be viewed as a temporal genotypic refuge of an ecosystem waiting a return to predisturbance conditions (Ellegaard, Godhe, & Riberio, 2018). Paleoecological data can help ecologists determine which taxa are appropriate for restored conditions (Wood, Perry, & Wilmshurst, 2017). With the ability to hatch, culture, and experiment with historical organisms from dormant propagules in lake sediments, resurrection ecology and paleolimnology allow insight into appropriate restoration goals and how a restored community might behave.

### 5.5 The challenges of marriage

The fields of paleoecology and resurrection ecology each have their own limitations that they bring to their “marriage”. Paleolimnologists often presume that a single core taken from a central deep basin provides all the information required to understand the ecological history of a lake. Biases associated with sediment cores and depositional environments are common and may include unconformable and hiatuses in sedimentation, violation of dating model assumptions, bioturbation, down‐core degradation and absence or loss of proxies, temporal resolution of sediment slices (i.e., details needed from each core slice vs. what is the period represented by that slice), and spatial biases in core records (e.g., littoral vs. profundal). Paleolimnologists are encouraged to exercise appropriate caution in coring, analyses, and interpretation (Battarbee et al., 2011) when considering temporal and spatial scaling of lake and landscape changes (Anderson, 2014).

For resurrection ecologists, one of the greatest limitations is the reduction in egg viability with sediment age, and furthermore, we do not how well the laboratory hatched propagules are representative of the overall diversity or dominant genotypes or phenotypes of the sampling period (Weis, 2018). In addition to the stratigraphic and geomorphic variability, we do not know how physical, chemical, or other limnological processes affect dormant propagule burial and preservation.

## 6 CONCLUSIONS

Lake sediments preserve unparalleled historical archives of organismal and environmental change. Paleoecologists use the power of multiproxy and multilake analyses to reconstruct precise records of historical change at lake, watershed, and global scales. New directions in paleolimnology are melding experimental neo‐ and paleo‐limnological approaches to better understand historical and future species and community response to environmental change. Resurrection ecologists rely on the “egg bank” of viable dormant propagules in lake sediments to test the capacity of organisms to adapt to our rapidly changing environment using (to date) *Daphnia* as the primary model organism. However, numerous aquatic organisms that are cornerstones in paleoecology (e.g., diatoms, cyanobacteria) also produce dormant propagules that offer new opportunities to test species and community adaptation, study eco‐evolutionary feedback and co‐evolution of primary producers and consumers, discern mechanisms of species response, and fuel the burgeoning collaboration between these two fields.
